# Enhancing growth, vitality, and aromatic richness: unveiling the dual magic of silicon dioxide and titanium dioxide nanoparticles in *Ocimum tenuiflorum* L.

**DOI:** 10.3389/fpls.2024.1335965

**Published:** 2024-02-06

**Authors:** Urooj Hassan Bhat, Moin Uddin, Aman Sobia Chishti, Sangram Singh, Sarika Singh, M. Masroor A. Khan, Mohammad Mukarram

**Affiliations:** ^1^ Advanced Plant Physiology Section, Department of Botany, Aligarh Muslim University, Aligarh, India; ^2^ Botany Section, Women’s College, Aligarh Muslim University, Aligarh, India; ^3^ Department of Phytology, Faculty of Forestry, Technical University in Zvolen, Zvolen, Slovakia

**Keywords:** essential oil, eugenol, holy basil, nanoparticles, nitrate reductase

## Abstract

*Ocimum tenuiflorum*, commonly known as “Holy basil,” is renowned for its notable medicinal and aromatic attributes. Its unique fragrance attributes to specific volatile phytochemicals, primarily belonging to terpenoid and/or phenylpropanoid classes, found within their essential oils. The use of nanoparticles (NPs) in agriculture has attracted attention among plant researchers. However, the impact of NPs on the modulation of morpho-physiological aspects and essential oil production in medicinal plants has received limited attention. Consequently, the present study aimed to explore the effect of silicon dioxide (SiO_2_) and titanium dioxide (TiO_2_) nanoparticles at various concentrations (viz., DDW (control), Si50+Ti50, Si100+Ti50, Si100+Ti100, Si200+Ti100, Si100+Ti200 and Si200+Ti200 mg L^-1^) on growth, physiology and essential oil production of *O. tenuiflorum* at 120 days after planting (DAP). The results demonstrated that the combined application of Si and Ti (Si100+Ti100 mg L^-1^) exhibited the most favourable outcomes compared to the other combinational treatments. This optimal treatment significantly increased the vegetative growth parameters (root length (33.5%), shoot length (39.2%), fresh weight (62.7%) and dry weight (28.5%)), photosynthetic parameters, enzymatic activities (nitrate reductase and carbonic anhydrase), the overall area of PGTs (peltate glandular trichomes) and essential oil content (172.4%) and yield (323.1%), compared to the control plants. Furthermore, the GCMS analysis showed optimal treatment (Si100+Ti100) significantly improved the content (43.3%) and yield (151.3%) of eugenol, the primary active component of the essential oil. This study uncovers a remarkable and optimal combination of SiO_2_ and TiO_2_ nanoparticles that effectively enhances the growth, physiology, and essential oil production in Holy basil. These findings offer valuable insights into maximizing the potential benefits of its use in industrial applications.

## 1 Introduction

Medicinal plants offer a promising avenue for promoting human health, given their accessibility and minimal side effects compared to expensive and potentially harmful synthetic drugs (Ahmad et al., 2020). Active phyto-compounds like coumarins, flavonoids, terpenes, carotenoids, amino acids and essential oils contribute to the therapeutic value of various plants (Ahmad et al., 2018). The Lamiaceae family of plants includes *Ocimum tenuiflorum*, which is native to Southeast Asia and is known as Tulsi in India. It is a fragrant shrub with a sweet and spicy scent (Olawale et al., 2022). *O. tenuiflorum* has been assigned a high level of importance by the National and State Medicinal Plant Boards due to its diverse range of therapeutic characteristics (George et al., 2021). *O. tenuiflorum* is used to cure a wide variety of ailments, including severe eye disorders, bronchial asthma, dysentery, skin diseases, bronchitis, persistent fever, arthritis, malaria, diarrhoea and insect bites (Patil et al., 2012). It is commonly used as an anti-inflammatory, antibacterial, and cardio-protective medication in medicine (Beltrán-Noboa et al., 2022).

The essential oils derived from Ocimum plants are complex combinations of organic compounds found in nature and exhibit numerous biological characteristics. The concentration and makeup of essential oils are influenced by the environmental factors, growth stage and maturity of the plant (Smitha and Tripathy, 2016). The volatile organic compounds present in the aerial parts of Ocimum are abundant in essential oils, which are highly sought after in the food and medicinal industries because of their valuable properties (Tangpao et al., 2022). During the synthesis procedure, the essential oil is biochemically produced and stored within specialised structures called glandular trichomes, which can be categorised into two distinct types: capitate glandular trichomes (CPTs) and peltate glandular trichomes (PGTs). These two types can be distinguished by the relative proportions and quantities of glandular cells (Oksanen, 2018; Gurav et al., 2022).

As a fast-growing interdisciplinary field of study, nanotechnology has many potential applications such as biomedicines, targeted drug delivery, wastewater treatment, agricultural and food packaging technologies, cancer therapy, electronics and biosensors, and the beauty industry (Tripathi et al., 2017; Saratale et al., 2018). The use of nanoparticles in agriculture aims to boost crop yields, reduce nutrient loss, and prevent disease in a sustainable manner. Nanomaterials have been found to affect fundamental aspects of plant development, including germination, photosynthesis, and yield (Khan et al., 2017). Recent findings have demonstrated that the utilisation of NPs, such as silicon dioxide (SiO_2_) and titanium dioxide (TiO_2_) nanoparticles, enhances both the quantity and quality of essential oils in *Vetiveria zizanioides* (Ahmed et al., 2022). This innovative application of nanotechnology presents a promising approach to unlock the complete potential of plants and address the challenges encountered by agricultural practitioners, as suggested by Ali et al. (2021).

Titanium dioxide nanoparticles (TiO_2_-NPs) are currently gaining significant attention due to their broad range of applications including antibacterial, antifungal, antiviral, anticancer, and antioxidative properties, as well as capabilities for drug delivery and various other medicinal uses (Sagadevan et al., 2022). Similarly, Andersen et al. (2016) demonstrated that the application of TiO_2_-NPs to *Brassica oleracea* improved plant growth and yield. In *O. basilicum*, the application of TiO_2_-NPs improved seed germination by enhancing plumule and radicle formation (Kiapour et al., 2015). TiO_2_-NPs treatment increased nutrient uptake and consequently improved the growth and development of plants (Fraceto et al., 2016). Single-generational exposure to nano-TiO_2_ modulates various plant processes, including germination, seedling growth, photosynthesis, metabolism, antioxidant defence machinery and yield (Siddiqi and Husen, 2017; Saratale et al., 2018; Sivakami et al., 2021). TiO_2_, predominantly in the nanoparticle (NP) form, is being extensively employed in various applications, thus necessitating a thorough evaluation of its potential implications for human health as well as aquatic and terrestrial ecosystems (Fiordaliso et al., 2022). On the other hand, various plant species are capable of accumulating huge quantities of silicon (Si), the second most prevalent element in soil crust (Chen et al., 2018). Silicon nanoparticles enter plants quickly and alter their metabolic processes due to their distinct physiological characteristics (Bansal et al., 2022). Because it is a long-term solution, the usage of Si and Si-NPs has become a popular agricultural practice (Rajput et al., 2021). The availability of silica NPs increased plant biomass while lowering crop metal consumption (Chen et al., 2018). Moreover, NPs application improved the antioxidant defences and mineral nutrient uptake, which ultimately increased the plant biomass when subjected to heavy metal stress (Hussain et al., 2018; Bidi et al., 2021). Thus, the multifaceted nature of SiO_2_ and TiO_2_-NPs has contributed to the growth, development, yield, and quality of aromatic and medicinal plants and are of great scientific significance and industrial applications.

Understanding the factors that influence the growth and essential oil content in Ocimum plants, including the impact of NPs, is crucial for optimising their cultivation and obtaining high-quality yields. Moreover, the combinational treatment approach of SiO_2_ and TiO_2_-NPs remains unexplored. We hypothesise that the combined treatment of SiO_2_ and TiO_2_-NPs will bring crop enhancement in terms of growth, development, and essential oil productivity. In this framework, the current study aims to establish a consistent method for applying these NPs to enhance plant development and the EO content and yield of *O. tenuiflorum*, offering added benefits for agronomists. This research can provide valuable insights of peltate glandular trichomes (PGTs) and the production of eugenol and cis-β-elemene, which are relevant to essential oil production in *O. tenuiflorum* plants.

## 2 Materials and methods

### 2.1 Growth conditions and plant material

The young plants of *Ocimum tenuiflorum* (Holy basil) were obtained from a local nursery in Aligarh. The experiment was conducted in the net house located at the Department of Botany, Aligarh Muslim University, Aligarh, under natural environmental conditions. Earthen pots with dimensions of 40 cm in diameter and 45 cm in height were used for the cultivation. Prior to planting, each pot was filled with a mixture of 8.5 kg of soil (Agricultural field, Department of Botany, AMU, Aligarh, **Supplementary File 1**) and farmyard organic manure. Random soil samples weighing 5.0 kg were collected from selected pots and sent to the Soil-Testing Laboratory at the Government Agriculture Farm, Quarsi, Aligarh, for analysis. The soil exhibited a sandy loam texture with a pH of 7.9 (**Supplementary File 1**). Before transplanting, recommended doses of N (in the form of urea), P (in the form of diammonium phosphate), and K (in the form of muriate of potash) at rates of 65.5, 92.3, and 54.0 mg kg^-1^ (soil), respectively, were applied. The experimental pots were kept under net house conditions with an average temperature of 29.24°C and a relative humidity of 70.32%.

### 2.2 Source of nanoparticles

SiO2 (Aerosil® 200) and TiO2 (Aeroxide® TiO2 P 90) nanoparticles were gifted from Evonik Industries (Germany). SiO2-NPs are a hydrophilic fumed silica with a specific surface area of 175–225 m^2^/g and a pH of about 3.7–4.5. However, TiO2-NPs are hydrophilic fumed metal oxide, bearing a specific surface area of about 70-110 m^2^/g and a pH of about 3.2–4.5.

### 2.3 Characterisation of NPs using electron microscopy

The structural characteristics and surface morphology of silicon dioxide (SiO_2_) and titanium dioxide (TiO_2_) nanoparticles were assessed utilising a scanning electron microscope (SEM) model JEOL JSM–6510 from Japan, operating at 20 kV. The SEM images were obtained across various levels of magnification, employing a secondary imaging detector to enable visualisation at the micrometre (μm) scale. Prior to analysis, the samples underwent gold coating at the University Sophisticated Instrumentation Facility (USIF), Aligarh Muslim University, Aligarh.

### 2.4 Experimental setup

In an experimental setup following a simple randomised design, solutions of SiO_2_-NPs and TiO_2_-NPs were prepared by dissolving in double distilled water (DDW) at varying concentrations (including DDW as control, Si50+Ti50, Si100+Ti50, Si100+Ti100, Si200+Ti100, Si100+Ti200, and Si200+Ti200 mg L^-1^). These specific combinations of treatments were selected based on a preliminary experiment provided as **Supplementary Material File 1**. A nursery of plants was made and after 30 days the plants were transferred to earthen pots. After 90 days of plantation (DAP), the plants were subjected to five spray treatments at a 5-day interval using a hand sprayer. Each treatment consisted of five replicates, with three plants in each replicate. After a growth period of 120 days, multiple parameters such as growth characteristics, physio-biochemical characteristics, essential oil content and yield, and active ingredient levels were evaluated.

### 2.5 Determination of growth attributes

The plant length (shoot and root) and biomass (fresh weight (FW) and dry weight (DW)) were measured after 120 days of transplant. Five plants from each treatment were carefully washed with tap water to remove any external debris and then dried using blotting paper. The root and shoot length was immediately measured using a metric scale. Afterwards, the plants were immediately weighed using digital balance to record the FW. The plants were incubated in hot-air oven at 80°C for 48 hours to determine the DW.

### 2.6 Determination of physiological and biochemical attributes

#### 2.6.1 Total chlorophyll content

The methodology outlined by Choudhary et al. (2020) was employed to quantify the overall chlorophyll concentrations within leaf samples. To initiate the analysis, 1g of freshly harvested leaf tissue was homogenised using 100% acetone. Subsequently, supernatant was collected following centrifugation at 10,000 rpm for 10 minutes. Optical density was measured at wavelengths of 663 nm and 645 nm to assess the levels of chlorophyll a and chlorophyll b. This was carried out utilising a Shimadzu UV-1700 spectrophotometer from Japan. The determination of total chlorophyll content was accomplished by summing the quantities of chlorophyll a and chlorophyll b. The results were expressed as mg g^-1^ FW.

#### 2.6.2 Chlorophyll fluorescence (Fv/Fm)

Saturation-Pulse Fluorometer (PAM-2000, Walz, Germany) was used to measure chlorophyll fluorescence (Fv/Fm) on the upper side of fully expanded leaves between 11:00 AM and 12:00 PM. The accurate readings were recorded by keeping the leaves in the dark for 30 minutes before measurement to stabilise the reaction centre. Low measuring beams with a light intensity of 125 mol m^2^ s^-1^ were used to determine the minimum (Fo) and maximum (Fm) fluorescence of dark-adapted leaves. The variable fluorescence (Fv) was calculated using the values of Fm − Fo and maximal efficiency of PSII (chlorophyll fluorescence) by using Fv/Fm.

#### 2.6.3 Carbonic anhydrase (CA) and nitrate reductase (NR) activities

Carbonic anhydrase (CA) activity in fresh leaves was measured using the protocol developed by Wani et al. (2023). Fresh leaf sample (0.2 g) devoid of veins were sliced into small rectangular pieces and submerged in 10 mL of 0.2 M aqueous cysteine hydrochloride solution. The reaction mixture was maintained at 4**°**C for 20 minutes. After incubation, the samples were transferred to a test tube containing 4 mL of a phosphate buffer (pH 6.8), 4 mL of a 0.2 M sodium bicarbonate (NaHCO_3_) solution, 0.2 mL of a 0.02 M sodium hydroxide (NaOH) solution, and 0.2 mL of 0.002% bromothymol-blue indicator. The reaction mixture was shaken for 20 seconds before being incubated in an ice box for 2 hours. Afterwards, the sample was titrated against 0.01 N HC1 using methylred as an indicator. The volume of HCl used to develop light purple colour was recorded. The CA activity was measured and expressed as mol CO_2_ kg**^-1^** leaf FW s**^-1^**.

Nitrate reductase (NR) activity was measured by the method pioneered by Jaworski (1971). 0.2 g of freshly diced leaves were transferred to a plastic vial with 0.5 mL of 0.2 M potassium nitrate solution, 2.5 mL of phosphate buffer (pH 7.5), and 2.5 mL of 5% isopropanol. After that, the reaction mixture was incubated for two hours using a BOD incubator maintained at 30 °C in dark. Post incubation, 0.4 mL of the reaction mixture was transferred to a test tube, followed by adding 0.3 mL each of 1% sulphanilamide and 0.02% N-(1-naphthyl) ethylenediamine dihydrochloride solution. After incubation at room temperature for 20 minutes to attain maximum colour intensity, the content was diluted with DDW to a final volume of 5 mL. Optical density of the final solution was measured at 540 nm using the spectrophotometer. NR activity was expressed as nmol (NO_2_) g**^−1^** FW h**^−1^**.

#### 2.6.4 Scanning electron microscopy (SEM) analysis

SEM examination was performed to visualise leaf ultra-structure, including stomata and trichomes. The leaves were placed in a 4% glutaraldehyde solution for 2 hours before being rinsed with a 0.1 M sodium cacodylate buffer solution with a pH of 7.2. Afterwards, the leaf samples were passed through the ethanol series (50, 70, 90 and 100%) and the dehydrated samples were viewed under SEM-JSM 6510 LV (JEOL, Japan).

### 2.7 Estimation of essential oil content by gas chromatography–mass spectrometry (GC-MS)

Fresh leaves (200 g) were collected from each treatment and chopped leaf-tissue was used to obtain essential oil (EO) using the hydro-distillation method. Leaf EO content was quantified gravimetrically in accordance with method of Ahmad et al. (2020). Clevenger’s apparatus was used to distil the chopped leaves for 3 hours, and the resulting EO was dehydrated with anhydrous sodium sulphate before being stored in sealed glass vials at 4°C until the GC analysis was carried out.

The US-made Agilent 7890B gas chromatography instrument was used for GC analysis. The instrument was equipped with a capillary column coated with fused silica carrying 30m length and 0.32 mm inner diameter in addition to an injector and flame ionisation detector. Nitrogen was employed as the carrier gas. Detector temperature 270°C, oven temperature 260°C, and injector temperature 250°C constituted the GC temperature profile. A sample size of 0.2 µL was used constantly. The first temperature was 40°C, maintained for 2 minutes, and the second and final temperature were 260°C, maintained for 10 minutes. Active components of EO, such as thymol and trans-caryophyllene, were identified using retention time. Content of EO and the active constituents was determined by comparing the chromatogram peaks of the sample to the peaks obtained from the reference standard according to Adams (2007).

## 3 Statistical analysis

The statistical analysis, including Two-way ANOVA, was conducted using software SPSS-23 (Chicago, USA) to assess significant differences. Duncan’s multiple range test (DMRT) was employed to determine significant variations at a significance level of P ≤ 0.05. The experiments were replicated five times, and the results are reported as the mean ± standard error (SE) for each measurement.

## 4 Results

Different parameters were studied to evaluate the effect of the combination of various concentrations of SiO_2_-NPs and TiO_2_-NPs on *Ocimum tenuiflorum*.

### 4.1 Characterisation of NPs using electron microscopy

**Figure 1:**
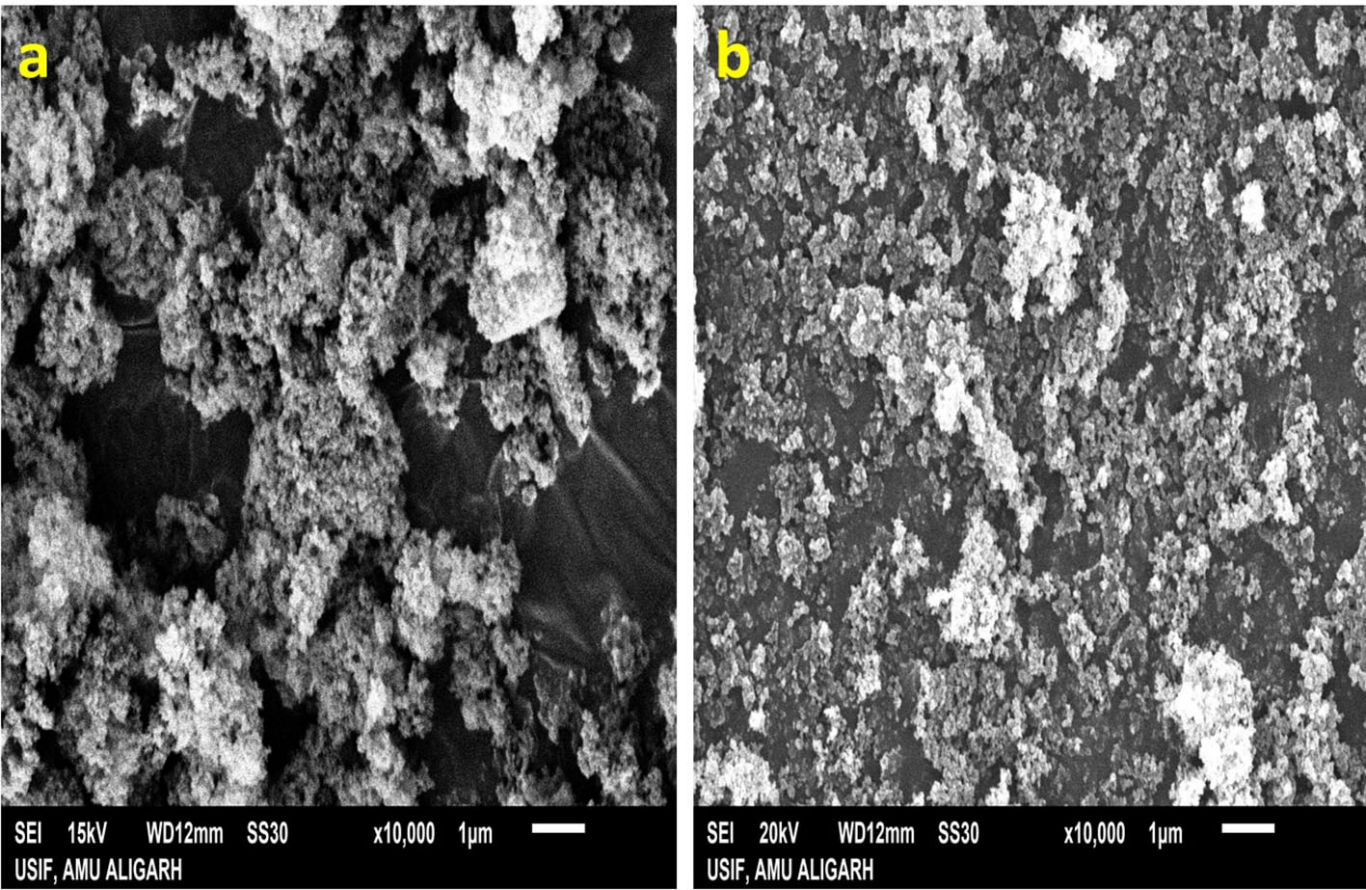
**(A)** SEM image of SiO_2_-NPs. **(B)** SEM image of TiO_2_-NPs.

The surface characteristics, morphology and size of SiO_2_ and TiO_2_-NPs were examined using scanning electron microscopy (SEM) techniques. The SEM images revealed the presence of spherical-shaped SiO_2_-NPs (**Figure 1A**), whereas knitted ball-like crystalline structure was observed in TiO_2_-NPs (**Figure 1B**). As clear from the figure, the average size of SiO_2_-NPs ranges between 5.2-12.1 nm and the average diameter of TiO_2_-NPs was about 10.2-15.2 nm.

### 4.2 Growth attributes

The foliar application impact of SiO_2_-NPs and TiO_2_-NPs increased roots and shoot length and plant fresh and dry weight, as compared to the control (**Supplementary Table 1**). Out of various treatments, the combined application of Si100+Ti100 mg L^-1^ excelled the other treatments and proved best in improving all the growth characteristics. The utmost increase of about 33.5% and 39.2% was noted in the root and shoot length and 62.7% and 28.5% in plant fresh and dry mass, respectively, over the control.

### 4.3 Physiological and biochemical attributes

#### 4.3.1 Photosynthetic attributes

**Figure 2:**
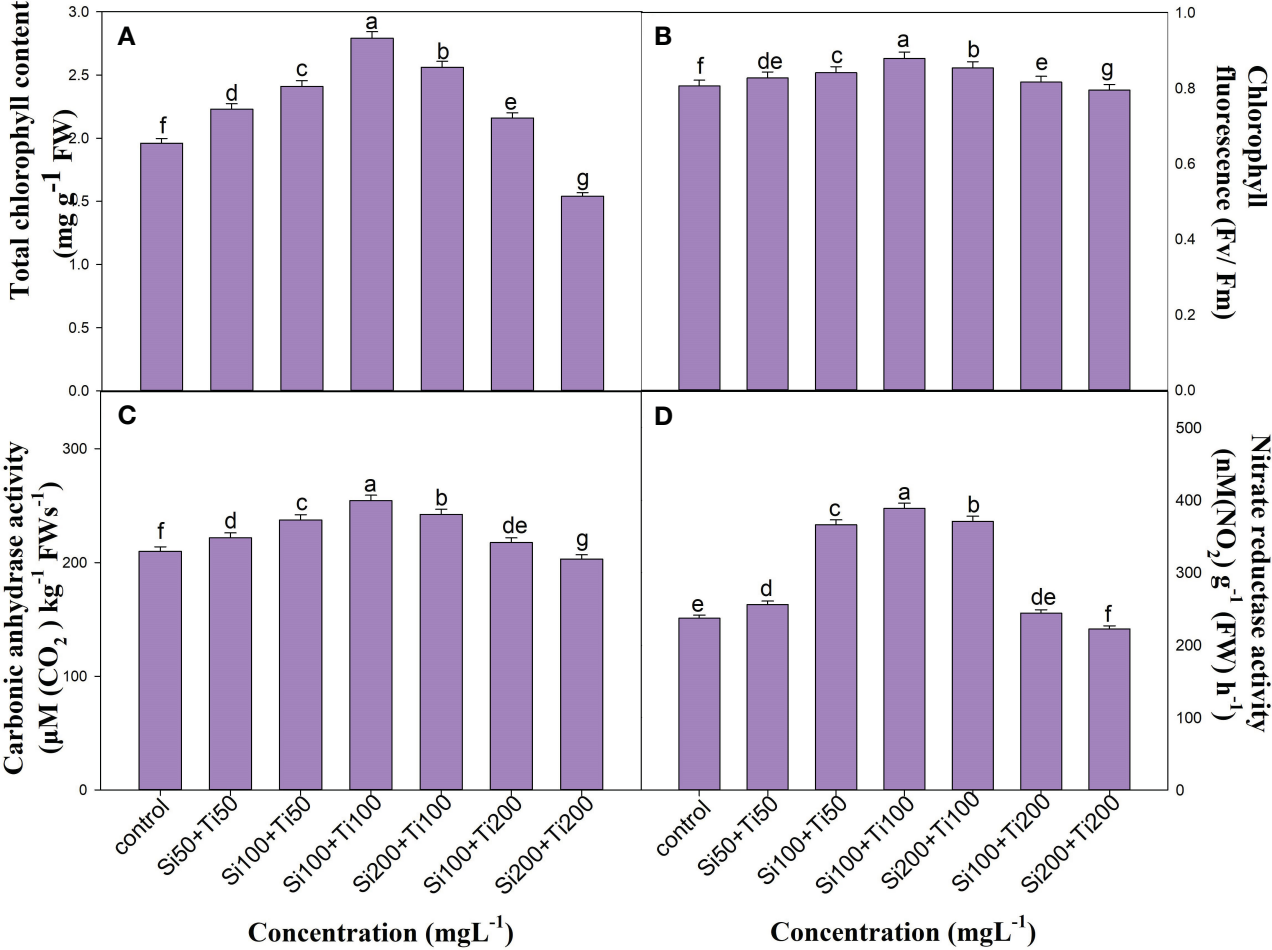
The effect of combined application of SiO_2_-NPs and TiO_2_-NPs on **(A)** Total chlorophyll content. **(B)** Chlorophyll fluorescence. **(C)** CA activity. **(D)** NR activity of *Ocimum tenuiflorum* recorded at 120 DAT. The small letter's indicate the significant differences among different treatments.

Foliar application of SiO_2_-NPs and TiO_2_-NPs improved photosynthetic parameters like total content of chlorophyll (TCC) and chlorophyll fluorescence (Fv/Fm). Values of all these parameters were increased up to 100 mg L^-1^ of SiO_2_-NPs and TiO_2_-NPs, but decreased at even higher concentrations. Among the various treatments, the combined application of Si100+Ti100 mg L^-1^ effectively increased the TCC and Fv/Fm by 42.3% and 9.05%, respectively (**Figure 2**).

#### 4.3.2 Carbonic anhydrase (CA) and nitrate reductase (NR) activities

The activities of CA and NR were shown to have increased after foliar application of SiO_2_-NPs and TiO_2_-NPs. Values of all these parameters were increased up to 100 mg L^-1^ concentration. After that, the values significantly decreased. The best treatment (Si100+Ti100 mg L^-1^) increased the CA and NR activity by 21.2% and 63.7%, respectively, over the control (**Figure 2**).

#### 4.3.3 Glandular trichome development

**Figure 3:**
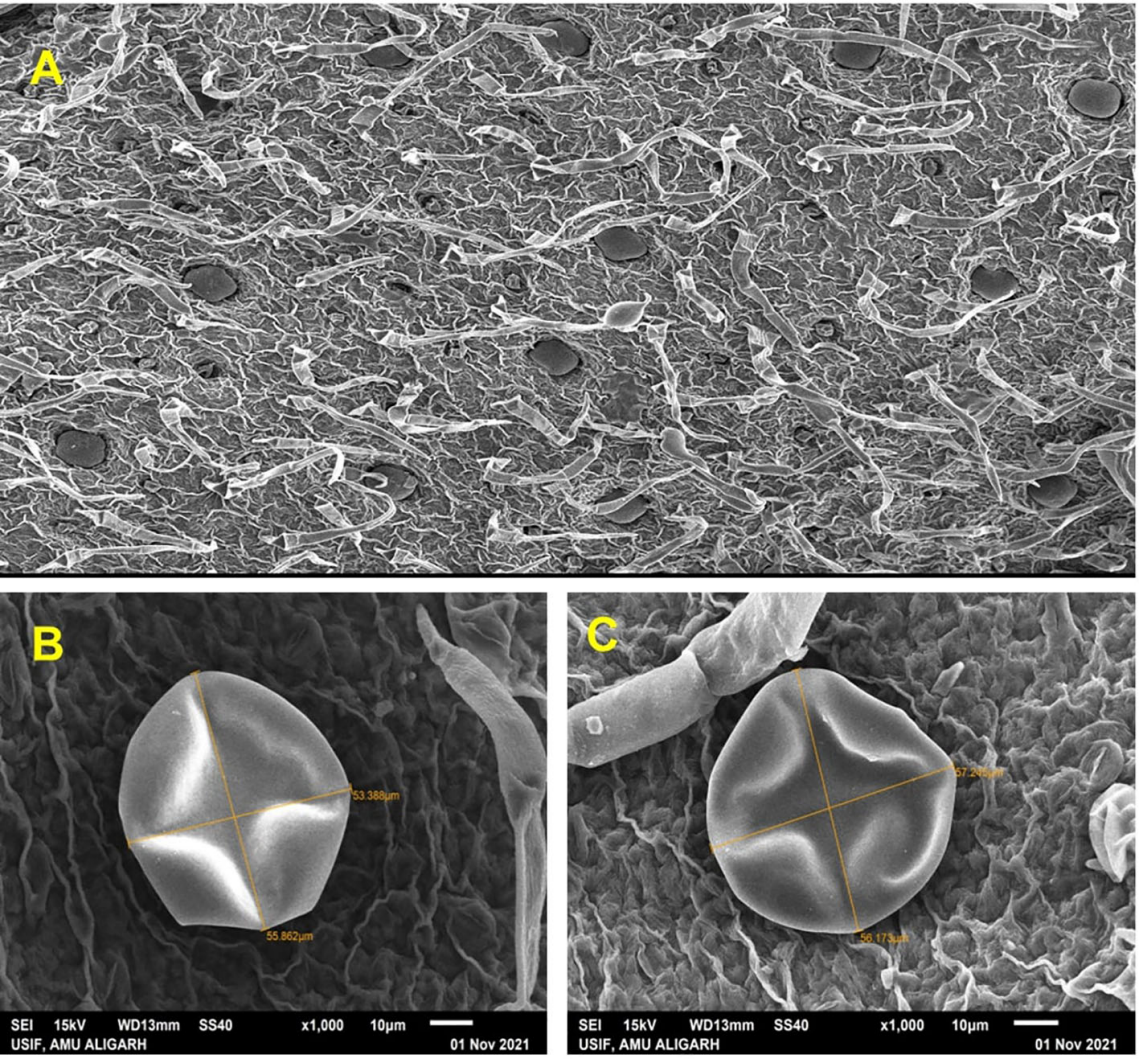
The effect of combined application of SiO_2_-NPs and TiO_2_-NPs on the glandular trichomes was determined using scanning electron microscopy (SEM). **(A)** Distribution of trichomes on leaf surface. **(B)** Control. **(C)** Treated (Si100+Ti100).

Our research found that foliar treatment resulted in a significant rise in peltate glandular trichome (PGT) area. The treatment at Si100+Ti100 mg L^-1^ concentrations had a considerable effect on the PGT area when compared to control. A maximum expansion of 3215.1cm^2^ was observed in the area of PGT after foliar application of NPs concentration in comparison to control (**Figure 3**).

#### 4.3.4 Essential oil content and yield

**Figure 4:**
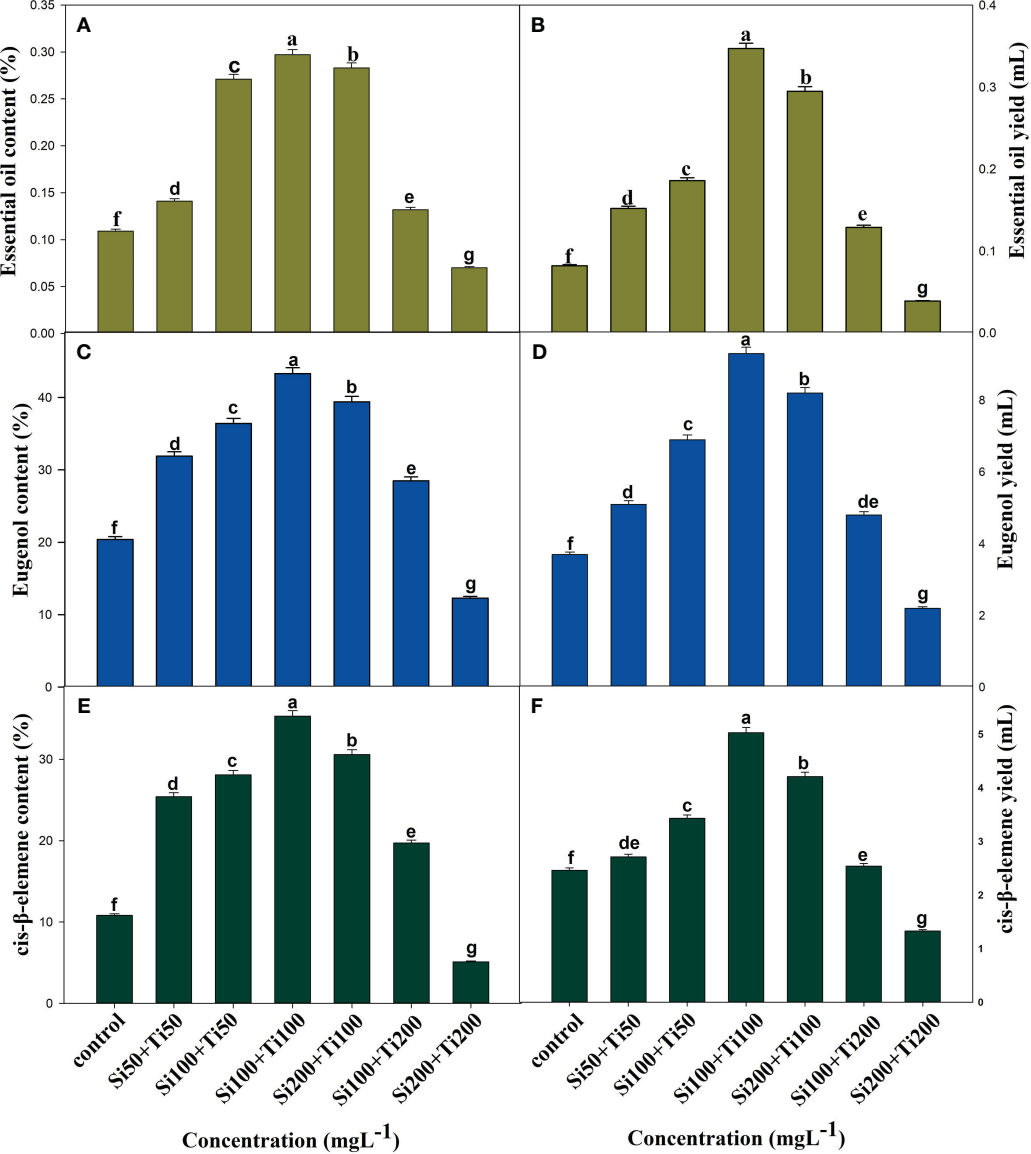
The Effects of combined application of SiO_2_-NPs and TiO_2_-NPs on essential oil of *O. tenuiflorum* at 120 DAP. **(A)** Essential oil content. **(B)** Essential oil yield per plant. **(C)** Eugenol content. **(D)** Eugenol yield. **(E)** Cis-β-elemene content. **(F)** Cis-β-elemene yield. The small letter's indicate the significant differences among different treatments.

Interestingly, the foliar application of SiO_2_-NPs and TiO_2_-NPs at 100 mg L^-1^ concentration optimally boosted the content and yield of EOs. The maximum percentage increase in content and yield of EO was seen by 172.4% and 323.1%, respectively, compared to the control. Furthermore, eugenol and cis-β-elemene content was significantly improved by 43.3% and 35.3% respectively, when compared to control plants (**Figure 4**).

## 5 Discussion

**Figure 5:**
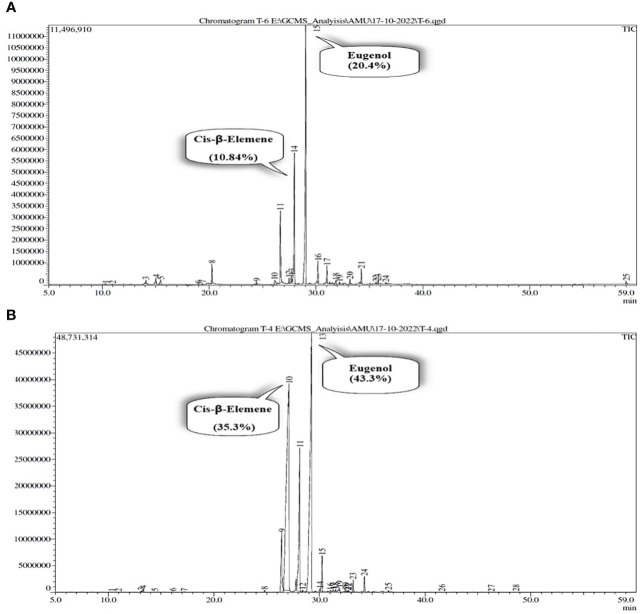
GCMS chromatogram demonstrates the identification and separation of components of essential oil in *Ocimum tenuiflorum*. **(A)** Double distilled water (DDW) treated plants (control). **(B)** Combined treatment of SiO_2_-NPs and TiO_2_-NPs at 100 mg L^-1^.

The application of growth-promoting substances has accelerated agricultural advancement, thereby enabling a partial realisation of the objective to enhance crop productivity (Ahmed et al., 2022). Nanoparticles (NPs) have attracted significant attention in agriculture for their elicitor properties and potential to enhance plant growth, thereby improving overall crop productivity (Mukarram et al., 2022; Uddin et al., 2023). Crop yields, nutrient loss, and disease prevention are all areas where nanomaterials could be utilised for betterment of agriculture. Some of the most essential stages of plant growth germination, photosynthesis, and flowering have been shown to be influenced by nanomaterials (Khan et al., 2017). The application of agrochemical sprays including fertilizers, nanoparticles, and elicitors, to the above-ground parts of plants is a globally recognised and effective agricultural practice (Shahhoseini and Daneshvar, 2023; Yin et al., 2023). Furthermore, bioavailability, concentration, solubility, and exposure time are various factors which influence the uptake and distribution of metallic nanoparticles throughout a plant (Uddin et al., 2023). Our findings demonstrate the significant impact of foliar application of SiO_2_-NPs and TiO_2_-NPs at various doses on the vegetative growth, physiology, and quality of *O. tenuiflorum*. The values of the all evaluated parameters showed an increase up to the concentration of Si100+Ti100 mg L^−1^. However, as the concentrations exceeded Si100+Ti100 mg L^−1^, there was a gradual decrease in the values (**Supplementary Table 1**; **Figures 2**–**5**). In particular significant enhancement in growth parameters was observed with the application of Si100+Ti100 mg L^−1^, as compared to control (**Supplementary Table 1**). Moreover, the physiological and biochemical characters exhibited a strong and favourable response to this specific concentration (**Figure 2**). Furthermore, the increase in chlorophyll content consequently enhanced photosynthetic efficiency, thereby enhancing growth, fresh and dry mass of the plants (Mir et al., 2022). Our study is supported by Ahmad et al. (2020), which stated that the plants treated with silicon improved their physical and biochemical defence mechanisms, resulting in effective germination and growth. Similar results were reported in *Brassica oleracea* (Anwar et al., 2023), *Brassica napus* (Huang et al., 2023) and *Ocimum basilicum* (Tan et al., 2018). Moreover, the observed enhancement in plant growth and development attributed to SiO_2_-NPs could potentially result from its influence on plant hormone and sugar metabolism (Maurya et al., 2019). TiO_2_ nanoparticles, on the other hand, have been found to exert substantial effects on the morphological, physiological, and biochemical traits of plants, which could contribute to an overall improvement in plant productivity (Gohari et al., 2020). Furthermore, the enhancement in fresh and dry weight of *Vetiveria zizanioides* was attributed to the combined application of SiO_2_ and TiO_2_-NPs (Li et al., 2023).

The foliar application of nanoparticles not only improved the growth but also enhances the photosynthetic attributes. For instance, Emamverdian et al. (2020), observed that the application of SiO_2_-NPs to Pb-stressed bamboo plants resulted a significant increase in chlorophyll content, which was associated with enhanced photosynthetic efficiency. In another study, the application of Si-NPs boosted plant growth and biomass yield by decreasing the oxidative stress in lemongrass (Mukarram et al., 2023) and wheat grains (Hussain et al., 2019). Moreover, SiO_2_ and TiO_2_-NPs have been found to improve the photosynthetic pigments in *Triticum aestivum* (Tripathi et al., 2016) and *Oryza sativa* (Rizwan et al., 2019; Zhang et al., 2020) plants and *Coleus aromaticus* (Uddin et al., 2023). Our findings, demonstrated a significant improvement in growth characteristics (**Supplementary Table 1**), photosynthetic pigments (**Figure 2**), and chlorophyll fluorescence (**Figure 2**) values mediated by combined treatment of SiO_2_ and TiO_2_-NPs. Moreover, the activities of carbonic anhydrase (CA) and nitrate reductase (NR) were maximally improved when SiO_2_ and TiO_2_-NPs were applied together at Si100+Ti100 concentration (**Figure 2**). These results are in the agreement with the widely held view that CA and NR activities are intimately linked to nitrogen and carbon metabolism, respectively, thereby directly contributing to photosynthesis, growth, and biomass production of the crop plants (Taiz et al., 2015). It has been revealed that the elevated Chl level has a stimulatory effect on photosynthesis, leading to an increase in the plant’s dry weight (DW) and fresh weight (FW) (Ahmad et al., 2020; Uddin et al., 2023). Our findings are consistent with the study conducted by Morteza et al. (2013), which reported similar observations regarding the positive effects of our treatments on growth parameters, enzyme activities, chloroplast activity (photosynthetic rate), and a decrease in chlorophyll degradation. These improvements resulted in enhanced CO_2_ assimilation and ultimately led to improved plant yield. The application of Si100+Ti100 mg L^-1^ through foliar spraying resulted in an enhancement of essential oil (EO) content and yield (**Figure 4**). These results are consistent with the findings reported by Cheng et al. (2009), who indicated that the production of secondary metabolites, including essential oils, is influenced by the equilibrium between carbohydrate source and sink. A higher ratio of source to sink promotes the synthesis of secondary metabolites, thereby increasing their production. Plants exposed to NPs showed elevated levels of photosynthetic pigments and carbonic anhydrase activity, suggesting that the plants were able to make more efficient use of the light and increase their carbohydrate content in the leaf (Khan et al., 2023). The biosynthesis of essential oils (EO) occurs in the chloroplasts through the Methyl Erythritol Phosphate (MEP) pathway, which is alternatively referred to as the non-mevalonate pathway. This pathway takes place within the plastids of the glandular trichomes’ secretory cells (Ahmad et al., 2020). The significant enlargement of the secretory glandular trichomes (PGTs) observed in this study is likely attributed to the accumulation of essential oils (EO), primarily terpenes, which are synthesised within these specialised structures using photosynthesis-derived products. As per GC-MS reports, application (**Figures 5A, B**) of SiO_2_ and TiO_2_-NPs demonstrated a significant increase in the concentration of Eugenol- a principal constituent of *O. tenuiflorum* (holy basil) essential oil, in comparison to control plants. The combined treatment of SiO_2_-NPs and TiO_2_-NPs at 100 mg L^-1^ significantly enhanced the content and yield of eugenol per plant.

Our study also found that the optimum treatment of Si100+Ti100 significantly affected the overall area of peltate glandular trichomes (PGT) in the leaves compared to the control (an increase of 3215.1cm^2^), which evidently might have resulted in high EO content of treated plants compared to those of the control (**Figure 4**). Conclusively, combined foliar application of Si_2_O and TiO_2_-NPs improves the quality of the essential oil by significantly enhancing the content and yield of Eugenol in the oil. In corroborations with our results Uddin et al. (2023) in *Coleus aromaticus*, Khan et al. (2023) in *Ocimum tenuiflorum*, and Ahmad et al. (2018) in *Mentha piperita* observed similar results by treating plants with different nanoparticles.

**Figure 6:**
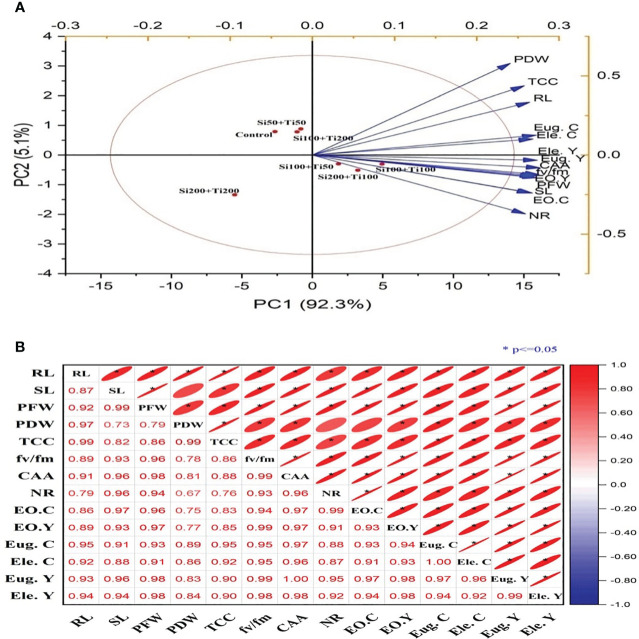
**(A)** Correlation analysis by principal component analysis. **(B)** Pearson correlation matrix. RL, root length; SL, shoot length; PFW, plant fresh weight; PDW, plant dry weight; TCC, total chlorophyll content; fv/fm, chlorophyll florescence; CAA, carbonic anhydrase activity; NR, nitrate reductase; EO.C, essential oil content; EO. Y, essential oil yield; Eug.C, eugenol content; Ele. C, elemene content; Eug.Y, eugenol yield; Ele.Y, elemene yield.

The correlation between the different parameters was analysed using principal component analysis (PCA) and a Pearson correlation matrix (**Figure 6**). The score and loading plot of PCA revealed a maximum (97.4%) variation among all the various parameters, with PC1 contributing 92.3% variation and PC2 displaying 5.1%. Significant positive correlations were found between various studied parameters, viz. plant dry weight, plant fresh weight, root length, shoot length, total chlorophyll content, chlorophyll florescence, carbonic anhydrase activity, nitrate reductase activity, essential oil content and yield. The PCA score and loading plot analysis revealed that a significant amount (97.4%) of the variation among the different parameters was observed. PC1 accounted for a substantial portion (92.3%) of the total variation, while PC2 contributed 5.1%. Furthermore, notable positive correlations were observed between the studied parameters including plant fresh weight, plant dry weight, root length, shoot length, total chlorophyll content, chlorophyll florescence, carbonic anhydrase and nitrate reductase activity, and content and yield of essential oil. In case of the score plot, the best treatment of Si100+Ti100at 100 mg L^-1^ had the greatest addition to PC1. Furthermore, the treatments like Si200+Ti100 and Si100+Ti50 showed a positive correlation with PC1. On the other hand, the treatments Si200+Ti200, Si100+Ti200, and Si50+Ti50, had no significant ameliorative impact.

## 6 Conclusion

Nanoparticles possess several advantageous characteristics, including cost-effectiveness, eco-friendliness, and water solubility, making them promising tools for improving plant growth and development. The present study emphasises the synergistic effect of SiO_2_ and TiO_2_ NPs, resulting in improved growth parameters, physio-biochemical attributes, photosynthetic efficiency and related enzymes, and enhanced yield in Holy basil. Additionally, the optimal treatment (Si100+Ti100 mg L^-1^) significantly improved the quality of essential oil due to the increasing the concentration of Eugenol. Therefore, current findings offer valuable insights into SiO_2_ and TiO_2_ NPs action in *O. tenuiflorum* plants to maximise crop economic benefits and their use in industrial and medicinal sectors.

## Data availability statement

The original contributions presented in the study are included in the article/**Supplementary Material**. Further inquiries can be directed to the corresponding author.

## Author contributions

UB: Data curation, Formal analysis, Investigation, Software, Writing – original draft. MU: Conceptualization, Project administration, Supervision, Validation, Writing – review & editing. AC: Software, Visualization, Writing – original draft. SanS: Software, Visualization, Writing – original draft. SarS: Software, Visualization, Writing – original draft. MMK: Project administration, Supervision, Writing – review & editing. MM: Funding acquisition, Writing – review & editing.

## References

[B1] Adams R. P. (2007). Identification of essential oil components by gas chromatography/mass spectrometry Vol. 456 (Carol Stream: Allured Publishing Corporation), 544–545.

[B2] Ahmad B. Khan M. Jaleel H. Shabbir A. Sadiq Y. Uddin M. (2020). Silicon nanoparticles mediated increase in glandular trichomes and regulation of photosynthetic and quality attributes in *Mentha piperita* L. J. Plant Growth Regul. 39 (1), 346–357. doi: 10.1007/s00344-019-09986-x

[B3] Ahmad B. Shabbir A. Jaleel H. Masroor M. Khan A. Sadiq Y. (2018). Efficacy of titanium dioxide nanoparticles in modulating photosynthesis, peltate glandular trichomes and essential oil production and quality in *Mentha piperita* L. Curr. Plant Biol. 13, 6–15. doi: 10.1016/j.cpb.2018.04.002

[B4] Ahmed K. B. M. Khan M. Shabbir A. Ahmad B. Uddin M. Azam A. (2022). Comparative effect of foliar application of silicon, titanium and zinc nanoparticles on the performance of vetiver-a medicinal and aromatic plant. Silicon. 15, 153–166. doi: 10.1007/s12633-022-02007-9

[B5] Ali M. Anjum N. Ain Q. T. He J. H. (2021). Homotopy perturbation method for the attachment oscillator arising in nanotechnology. Fibers Polymers 22, 1601–1606. doi: 10.1007/s12221-021-0844-x

[B6] Andersen C. P. King G. Plocher M. Storm M. Pokhrel L. R. Johnson M. G.. (2016). Germination and early plant development of ten plant species exposed to titanium dioxide and cerium oxide nanoparticles. Environ. Toxicol. Chem. 35(9), 2223–2229. doi: 10.1002/etc.3374 26773270

[B7] Anwar T. Qureshi H. Fatimah H. Siddiqi E. H. Anwaar S. Moussa I. M.. (2023). Improvement of physio-biochemical attributes and mitigation of salinity stress by combined application of melatonin and silicon nanoparticles in *Brassica oleracea* var. botrytis. Scientia Hortic. 322, 112456. doi: 10.1016/j.scienta.2023.112456

[B8] Bansal K. Hooda V. Verma N. Kharewal T. Tehri N. Dhull V.. (2022). Stress alleviation and crop improvement using silicon nanoparticles in agriculture: a review. Silicon 14, 1–14. doi: 10.1007/s12633-022-01755-y

[B9] Beltrán-Noboa A. Proaño-Ojeda J. Guevara M. Gallo B. Berrueta L. A. Giampieri F.. (2022). Metabolomic profile and computational analysis for the identification of the potential anti-inflammatory mechanisms of action of the traditional medicinal plants *Ocimum basilicum* and *Ocimum tenuiflorum* . Food Chem. Toxicol. 164, 113039. doi: 10.1016/j.fct.2022.113039 35461962

[B10] Bidi H. Fallah H. Niknejad Y. BarariTari D. (2021). Iron oxide nanoparticles alleviate arsenic phytotoxicity in rice by improving iron uptake, oxidative stress tolerance and diminishing arsenic accumulation. Plant Physiol. Biochem. 163, 348–357. doi: 10.1016/j.plaphy.2021.04.020 33915441

[B11] Chen R. Zhang C. Zhao Y. Huang Y. Liu Z. (2018). Foliar application with nano-silicon reduced cadmium accumulation in grains by inhibiting cadmium translocation in rice plants. Environ. Sci. pollut. Res. 25, 2361–2368. doi: 10.1007/s11356-017-0681-z 29124638

[B12] Cheng S. Y. Xu F. Wang Y. (2009). Advances in the study of flavonoids in *Ginkgo biloba* leaves. J. Med. Plants Res. 3 (13), 1248–1252. Available at: https://api.semanticscholar.org/CorpusID:82865179.

[B13] Choudhary S. Zehra A. Naeem M. Khan M. M. A. Aftab T. (2020). Effects of boron toxicity on growth, oxidative damage, antioxidant enzymes and essential oil fingerprinting in *Mentha arvensis* and *Cymbopogon flexuosus* . Chem. Biol. Technol. Agric. 7, 1–11. doi: 10.1186/s40538-019-0175-y

[B14] Emamverdian A. Ding Y. Mokhberdoran F. Xie Y. Zheng X. Wang Y. (2020). Silicon dioxide nanoparticles improve plant growth by enhancing antioxidant enzyme capacity in bamboo (*Pleioblastus pygmaeus*) under lead toxicity. Trees. 34 (2), 469–481. doi: 10.1007/s00468-019-01929-z

[B15] Fiordaliso F. Bigini P. Salmona M. Diomede L. (2022). Toxicological impact of titanium dioxide nanoparticles and food-grade titanium dioxide (E171) on human and environmental health. Environ. Sci. Nano. 9 (4), 1199–1211. doi: 10.1039/D1EN00833A

[B16] Fraceto L. F. Grillo R. de Medeiros G. A. Scognamiglio V. Rea G. Bartolucci C. (2016). Nanotechnology in agriculture: which innovation potential does it have? Sci. 4. doi: 10.3389/fenvs.2016.00020

[B17] George D. Sindhu P. V. Menon M. V. (2021). Effect of harvesting time and height of harvest on the performance of tulsi (Ocimum tenuiflorum L.) under shade and open condition. J. Med. Plant Res. 9 (2), 110–114. doi: 10.1038/s41598-020-57794-1

[B18] Gohari G. Mohammadi A. Akbari A. Panahirad S. Dadpour M. R. Fotopoulos V.. (2020). Titanium dioxide nanoparticles (TiO_2_ NPs) promote growth and ameliorate salinity stress effects on essential oil profile and biochemical attributes of *Dracocephalum moldavica* . Sci. Rep. 10, 912. doi: 10.1038/s41598-020-57794-1 31969653 PMC6976586

[B19] Gurav T. P. Dholakia B. B. Giri A. P. (2022). A glance at the chemodiversity of *Ocimum* species: Trends, implications, and strategies for the quality and yield improvement of essential oil. Phytochem. Rev. 21, 879–913. doi: 10.1007/s11101-021-09767-z 34366748 PMC8326315

[B20] Huang Q. Ayyaz A. Farooq M. A. Zhang K. Chen W. Hannan F.. (2023). Silicon dioxide nanoparticles enhance plant growth, photosynthetic performance, and antioxidants defence machinery through suppressing chromium uptake in *Brassica napus* L. Environ. pollut. 342, 123013. 10.1016/j.envpol.2023.123013 38012966

[B21] Hussain A. Ali S. Rizwan M. Rehman M. Z. Javed M. R. Imran M.. (2018). Zinc oxide nanoparticles alter the wheat physiological response and reduce the cadmium uptake by plants. Environ. pollut. 242, 1518–1526. doi: 10.1016/j.envpol.2018.08.036 30144725

[B22] Hussain A. Rizwan M. Ali Q. Ali S. (2019). Seed priming with silicon nanoparticles improved the biomass and yield while reduced the oxidative stress and cadmium concentration in wheat grains. Environ. Sci. pollut. Res. 26, pp.7579–7588. doi: 10.1007/s11356-019-04210-5 30661166

[B23] Jaworski E. G. (1971). Nitrate reductase assay in intact plant tissues. Biochem. Biophys. Res. Commun. 43 (6), 1274–1279. doi: 10.1016/S0006-291X(71)80010-4 5106073

[B24] Khan M. M. A. Quasar N. Afreen R. (2023). Nanotized form of indole acetic acid improve biochemical activities, the ultrastructure of glandular trichomes and essential oil production in *Ocimum tenuiflorum* L. Ind. Crops Products 193, p.116117. doi: 10.1016/j.indcrop.2022.116117

[B25] Khan M. N. Mobin M. Abbas Z. K. Al Mutairi K. A. Siddiqui Z. H. (2017). Role of nanomaterials in plants under challenging environments. Plant Physiol. Biochem. 110, 194–209. doi: 10.1016/j.plaphy.2016.05.038 27269705

[B26] Kiapour H. Moaveni P. Habibi D. (2015). Evaluation of the application of gibbrellic acid and titanium dioxide nanoparticles under drought stress on some traits of basil (*Ocimum basilicum* L.). Int. j. agron. agric., 138–150.

[B27] Li Y. Xi K. Liu X. Han S. Han X. Li G.. (2023). Silica nanoparticles promote wheat growth by mediating hormones and sugar metabolism. J. Nanobiotechnology 21 (1), p.2. doi: 10.1186/s12951-022-01753-7 PMC9808955 36593514

[B28] Maurya S. Chandra M. Yadav R. K. Narnoliya L. K. Sangwan R. S. Bansal S.. (2019). Interspecies comparative features of trichomes in Ocimum reveal insights for biosynthesis of specialized essential oil metabolites. Protoplasma 256 (4), 893–907. doi: 10.1007/s00709-018-01338-y 30656458

[B29] Mir A. R. Alam P. Hayat S. (2022). Perspective of melatonin-mediated stress resilience and Cu remediation efficiency of *Brassica juncea* in Cu-contaminated soils. Front. Plant Sci. 13, 910714. doi: 10.3389/fpls.2022.910714 35923886 PMC9340790

[B30] Morteza E. Moaveni P. Farahani H. A. Kiyani M. (2013). Study of photosynthetic pigments changes of maize (*Zea mays* L.) under nano TiO_2_ spraying at various growth stages. Springer Plus. 2 (1), 1–5. doi: 10.1186/2193-1801-2-247 23847752 PMC3696182

[B31] Mukarram M. Khan M. M. A. Kurjak D. Lux A. Corpas F. J. (2023). Silicon nanoparticles (SiNPs) restore photosynthesis and essential oil content by upgrading enzymatic antioxidant metabolism in lemongrass (*Cymbopogon flexuosus*) under salt stress. Front. Plant Sci. 14, 1116769. doi: 10.3389/fpls.2023.1116769 36875580 PMC9981966

[B32] Mukarram M. Petrik P. Mushtaq Z. Khan M. M. A. Gulfishan M. Lux A. (2022). Silicon nanoparticles in higher plants: Uptake, action, stress tolerance, and crosstalk with phytohormones, antioxidants, and other signalling molecules. Environ. pollut. 310, 119855. doi: 10.1016/j.envpol.2022.119855 35940485

[B33] Oksanen E. (2018). Trichomes form an important first line of defence against adverse environment—new evidence for ozone stress mitigation. Plant Cell Environ. 41 (7), 1497–1499. doi: 10.1111/pce.13187 29508922

[B34] Olawale F. Ariatti M. Singh M. (2022). *Ocimum tenuiflorum* L. mediated green synthesis of silver and selenium nanoparticles: antioxidant activity, cytotoxicity and density functional theory studies. Adv. Nat. Sci.: Nanosci. Nanotechnol. 13(1) 015015. doi: 10.1088/2043-6262/ac5d4a

[B35] Patil R. S. Kokate M. R. Kolekar S. S. (2012). Bioinspired synthesis of highly stabilized silver nanoparticles using *Ocimum tenuiflorum* leaf extract and their antibacterial activity. Spectrochim. Acta part A: mol Biomol. Spectrosc. 91, 234–238. doi: 10.1016/j.saa.2012.02.009 22381796

[B36] Rajput V. D. Minkina T. Feizi M. Kumari A. Khan M. Mandzhieva S.. (2021). Effects of silicon and silicon-based nanoparticles on rhizosphere microbiome, plant stress and growth. Biology. 10 (8), 791. doi: 10.3390/biology10080791 34440021 PMC8389584

[B37] Rizwan M. Ali. S. urRehman M. Z. Malik S. Adrees M. Qayyum M. F.. (2019). Effect of foliar applications of silicon and titanium dioxide nanoparticles on growth, oxidative stress, and cadmium accumulation by rice (*Oryza sativa*). Acta Physiol. Plant 41, 35. doi: 10.1007/s11738-019-2828-7

[B38] Sagadevan S. Imteyaz S. Murugan B. Lett J. A. Sridewi N. Weldegebrieal G. K.. (2022). A comprehensive review on green synthesis of titanium dioxide nanoparticles and their diverse biomedical applications. Green Process. Synth. 11(1), 44–63. doi: 10.1515/gps-2022-0005

[B39] Saratale R. G. Karuppusamy I. Saratale G. D. Pugazhendhi A. Kumar G. Park Y.. (2018). A comprehensive review on green nanomaterials using biological systems: Recent perception and their future applications. Colloids Surfaces B: Biointerfaces 170, 20–35. doi: 10.1016/j.colsurfb.2018.05.045 29860217

[B40] Shahhoseini R. Daneshvar H. (2023). Phytochemical and physiological reactions of feverfew (*Tanacetum parthenium* (L.) Schultz Bip) to TiO_2_ nanoparticles. Plant Physiol. Biochem. 194, 674–684. doi: 10.1016/j.plaphy.2022.12.011 36563573

[B41] Siddiqi K. S. Husen A. (2017). Plant response to engineered metal oxide nanoparticles. Nano. Res. Lett. 12 (1), 92. doi: 10.1186/s11671-017-1861-y PMC5293712 28168616

[B42] Sivakami A. Sarankumar R. Vinodha S. (2021). Introduction to nanobiotechnology: novel and smart applications. Bio-manufactured Nanomaterials: Perspect. Promotion, 1–22. doi: 10.1007/978-3-030-67223-2_1

[B43] Smitha G. R. Tripathy V. (2016). Seasonal variation in the essential oils extracted from leaves and inflorescence of different *Ocimum* species grown in Western plains of India. Ind. Crops Prod. 94, 52–64. doi: 10.1016/j.indcrop.2016.07.041

[B44] Taiz L. Zeiger E. Moller I. M. Murphy A. (2015). Plant physiology and development. 6th Edition (Sunderland, CT: Sinauer Associates).

[B45] Tan W. Du W. Darrouzet-Nardi A. J. Hernandez-Viezcas J. A. Ye Y. Peralta-Videa J. R.. (2018). Effects of the exposure of TiO_2_ nanoparticles on basil (*Ocimum basilicum*) for two generations. Sci. Total Environ. 636, 240–248. doi: 10.1016/j.scitotenv.2018.04.263 29705436

[B46] Tangpao T. Charoimek N. Teerakitchotikan P. Leksawasdi N. Jantanasakulwong K. Rachtanapun P.. (2022). Volatile organic compounds from basil essential oils: plant taxonomy, biological activities, and their applications in tropical fruit productions. Horticulturae. 8 (2), 144. doi: 10.3390/horticulturae8020144

[B47] Tripathi D. K. Singh S. Singh V. P. Prasad S. M. Chauhan D. K. Dubey N. K. (2016). Silicon nanoparticles more efficiently alleviate arsenate toxicity than silicon in maize cultiver and hybrid differing in arsenate tolerance. Front. Environ. Sci. 4. doi: 10.3389/fenvs.2016.00046

[B48] Tripathi D. K. Singh S. Singh V. P. Prasad S. M. Dubey N. K. Chauhan D. K. (2017). Silicon nanoparticles more effectively alleviated UV-B stress than silicon in wheat (*Triticum aestivum*) seedlings. Plant Physiol. Biochem. 110, 70–78. doi: 10.1016/j.plaphy.2016.06.026 27470120

[B49] Uddin M. Bhat U. H. Singh S. Singh S. Chishti A. S. Khan M. M. A. (2023). Combined application of SiO_2_ and TiO_2_ nanoparticles enhances growth characters, physiological attributes and essential oil production of *Coleus aromatics* Benth. Heliyon 9 (11), e21646. doi: 10.1016/j.heliyon.2023.e21646 38058652 PMC10695843

[B50] Wani K. I. Zehra A. Choudhary S. Naeem M. Khan M. M. A. Khan R.. (2023). Exogenous strigolactone (GR24) positively regulates growth, photosynthesis, and improves glandular trichome attributes for enhanced artemisinin production in *Artemisia annua* . J. Plant Growth Regul. 42 (8), 4606–4615. doi: 10.1007/s00344-022-10654-w PMC8993037 35431419

[B51] Yin J. Su X. Yan S. Shen J. (2023). Multifunctional nanoparticles and nanopesticides in agricultural application. Nanomaterials 13 (7), 1255. doi: 10.3390/nano13071255 37049348 PMC10096623

[B52] Zhang W. Long J. Geng J. Li J. Wei Z. (2020). Impact of titanium dioxide nanoparticles on Cd phytotoxicity and bioaccumulation in rice (*Oryza sativa* L.). Int. J. Environ. Res. Public Health 17 (9), 2979. doi: 10.3390/ijerph17092979 32344831 PMC7246507

