# Supplementary material for: Enhancing growth, vitality, and aromatic richness: unveiling the dual magic of silicon dioxide and titanium dioxide nanoparticles in Ocimum tenuiflorum L
Source: Front Plant Sci. 2024 Feb 6;15:1335965. doi: 10.3389/fpls.2024.1335965 (PMC10880381; doi:10.3389/fpls.2024.1335965)
Supplement: Supplementary file 1 [file Table_1.docx]

**Table 1. Effect of foliar application of Silicon-dioxide nanoparticles on growth parameters of *Ocimum tenuiflorum* recorded at 90 DAT (days after transplanting).**

| **Treatments** | **Root length**  **(cm)**  **(mean±SE)** | **Shoot length (cm)**  **(mean±SE)** | **Root**  **(FW)**  **(g)** | **Root**  **(DW)**  **(g)** | **Shoot**  **(FW)** | **Shoot**  **(DW)** |
| --- | --- | --- | --- | --- | --- | --- |
| **Control**  **(DDW)** | 20.33±0.3^d^ | 35.32±0.6^d^ | 40.51±0.7^d^ | 17.25±0.3^d^ | 98.65±1.8^d^ | 46.32±0.8^d^ |
| **50** | 21.26±0.4^c^ | 37.43±0.7^c^ | 48.97±0.9^c^ | 22.56±0.4^c^ | 110.46±2.1^c^ | 52.23±0.9^c^ |
| **100** | 29.22±0.5^a^ | 46.19±0.8^a^ | 68.35±1.3^a^ | 31.15±0.5^a^ | 134.45±2.5^a^ | 64.22±1.2^a^ |
| **200** | 26.53±0.5^b^ | 41.33±0.7^b^ | 52.43±0.9^b^ | 23.21±0.4^b^ | 112.21±2.1^b^ | 55.16±1.0^b^ |
| **400** | 16.04±0.3^e^ | 33.62±0.6^e^ | 37.59±0.7^e^ | 15.73±0.2^e^ | 87.55±1.6^e^ | 40.77±0.7^e^ |

**Conclusion:** Among various doses of SiO_2_-NPs, plants respond differently in a dose dependent manner and it was observed that application of 50 and 100 mgL^-1^ of SiO_2_-NPs positively influenced the plant growth (order of beneficial response was 50 and 100 mgL^-1^), however, application of 200 and 400 mgL^-1^ of SiO_2_-NPs negatively influenced the plant growth.

**Table 2. Effect of foliar application of Titanium-dioxide nanoparticles on growth parameters of *Ocimum tenuiflorum* recorded at 90 DAT (days after transplanting).**

| **Treatments** | **Root length**  **(cm)**  **(mean±SE)** | **Shoot length (cm)**  **(mean±SE)** | **Root**  **(FW)**  **(g)** | **Root**  **(DW)**  **(g)** | **Shoot**  **(FW)** | **Shoot**  **(DW)** |
| --- | --- | --- | --- | --- | --- | --- |
| **Control**  **(DDW)** | 22.42±0.4^d^ | 37.23±0.7^d^ | 42.61±0.8^d^ | 19.32±0.3^d^ | 101.16±1.9^d^ | 48.18±0.9^d^ |
| **50** | 24.27±0.4^c^ | 40.15±0.7^c^ | 44.24±0.8^c^ | 20.32±0.3^c^ | 104.13±1.9^c^ | 45.22±0.8^c^ |
| **100** | 35.53±0.6^a^ | 56.21±1.0^a^ | 70.33±1.3^a^ | 32.15±0.6^a^ | 136.15±2.5^a^ | 66.32±1.2^a^ |
| **200** | 32.11±0.6^b^ | 44.12±0.8^b^ | 54.42±1.0^b^ | 25.24±0.4^b^ | 114.22±2.1^b^ | 53.21±1.0^b^ |
| **400** | 19.39±0.3^e^ | 34.28±0.6^e^ | 37.15±0.7^e^ | 16.51±0.3^e^ | 84.25±1.6^e^ | 33.42±0.6^e^ |

**Conclusion:** Among various doses of TiO_2_-NPs, plants respond differently in a dose dependent manner and it was observed that application of 50 and 100 mg L^-1^ of TiO_2_-NPs positively influenced the plant growth (order of beneficial response was 50 and 100 mgL^-1^), however, application of 200 and 400 mgL^-1^ of TiO_2_-NPs negatively influenced the plant growth.

**Table 3: Physio-chemical properties of soil**

| **Characteristics** | **Soil** |
| --- | --- |
| Texture | Sandy loam |
| pH | 7.9 ± 0.2 |
| NO_3_^-^ (mg kg^−1^ soil) | 152.7 ± 0.015 |
| P (mg kg^−1^ soil) | 44.3 ± 0.010 |
| K (mg kg^−1^ soil) | 156.1 ± 1.06 |
| Ca (mg kg^−1^ soil) | 16.35 ± 0.95 |
| Mg (mg kg^−1^ soil) | 28.80 ± 1.02 |
| Cl^-^ (mg kg^−1^ soil) | 21.90 ± 1.22 |
| Na (mg kg^−1^ soil) | 10.10 ± 0.43 |
| SO_4_^2-^ (mg kg^−1^ soil) | 12.72± 0.40 |
| HCO_3_ (mg kg^−1^ soil) | 15.95 ± 0.40 |
| CO_3_^2-^ (mg kg^−1^ soil) | 67.61 ± 0.96 |
| Cu (mg kg^−1^ soil) | 12.0± 0.40 |
